# Discoloration of surface sealants by plaque disclosing solution

**DOI:** 10.1007/s00056-020-00227-5

**Published:** 2020-05-06

**Authors:** Sinan Şen, Ralf Erber, Gözde Şen, Nadine Deurer, Sebastian Zingler, Christopher J. Lux

**Affiliations:** 1 Department of Orthodontics and Dentofacial Orthopaedics, Dental School, University of Heidelberg, Im Neuenheimer Feld 400, 69120 Heidelberg, Germany; 2 MVZ Dentale Praxisklinik, Dr. Dilling & Kollegen GmbH, Fleiner Straße 3, 74072 Heilbronn, Germany

**Keywords:** Orthodontic treatment, Discoloration, Surface sealants, Staining, Tooth cleaning, Kieferorthopädische Behandlung, Verfärbung, Glattflächenversiegler, Anfärben, Zahnreinigung

## Abstract

**Purpose:**

Surface sealants are widely used as a prevention strategy and are indicated for young patients with insufficient oral hygiene who also need plaque removal by professional tooth cleaning. The aim of this study was to evaluate discoloration of surface sealants by plaque disclosing solutions and to test to what extent this discoloration can be reduced again by professional tooth cleaning.

**Methods:**

In all, 96 extracted lesion-free human teeth were randomly assigned to treatment with either Pro Seal® (PS; Opal Orthodontics, South Jordan, UT, USA) or Opal®Seal™ (OS; Reliance Orthodontic Products, Itasca, IL, USA). Color evaluations after application of the plaque disclosing solution Mira-2-Ton® (Hager & Werken, Duisburg, Germany) were performed using a clinical spectrophotometer. Staining and polishing were repeated once. Color differences (∆E) above 3.77 were regarded as clinically relevant.

**Results:**

All sealants showed high, clinically relevant ∆E values after the first staining. Polishing led to significantly decreased ∆E values on PS-treated teeth; however, the median ∆E value remained above the clinically relevant threshold. Polishing on OS-treated teeth only slightly reduced ∆E values. After professional tooth cleaning both PS and OS showed clinically relevant ∆E values.

**Conclusion:**

Surface sealants show clinically relevant discoloration after exposure to plaque disclosing solution under in vitro conditions. Such discolorations could not be removed by professional tooth cleaning. Thus, in clinical practice, plaque disclosing solutions might cause esthetic deficits in surface sealant-treated teeth. The impact of plaque disclosing solutions under clinical conditions (e.g., in the presence of saliva and by various aspects of a person’s nutrition) should be investigated in clinical studies.

## Abstract (de)

**Zielsetzung:**

Glattflächenversiegler werden häufig als Präventionsstrategie eingesetzt und sind für junge Patienten mit unzureichender Mundhygiene indiziert, die auch Plaqueentfernung durch professionelle Zahnreinigung benötigen. Ziel der Studie war es, die Verfärbung von Glattflächenversieglern durch Plaquefärbelösung zu evaluieren und zu prüfen, inwieweit diese Verfärbung durch professionelle Zahnreinigung wieder reduziert werden kann.

**Methoden:**

Insgesamt 96 extrahierte läsionsfreie humane Zähne wurden nach dem Zufallsprinzip der Behandlung mit Pro Seal® (PS; Opal Orthodontics, South Jordan, UT, USA) oder Opal®Seal^TM^ (OS; Reliance Orthodontic Products, Itasca, IL, USA) zugeordnet. Die Farbbestimmungen nach Anwendung der Mira-2-Ton® (Hager & Werken, Duisburg, Deutschland) Plaquefärbelösung wurden mit einem klinischen Spektrophotometer durchgeführt. Färbung und Politur wurden noch einmal wiederholt. Farbunterschiede (∆E) über 3,77 wurden als klinisch relevant angesehen.

**Ergebnisse:**

Alle Versiegelungen zeigten hohe, klinisch relevante ∆E-Werte nach der ersten Färbung. Das Polieren führte zu signifikant verringerten ∆E-Werten bei den mit PS behandelten Zähnen; der Medianwert von ∆E blieb jedoch über dem klinisch relevanten Schwellenwert. Das Polieren auf OS-behandelten Zähnen reduzierte die ∆E-Werte nur geringfügig. Nach der professionellen Zahnreinigung zeigten sowohl PS als auch OS klinisch relevante ∆E-Werte.

**Schlussfolgerungen:**

Glattflächenversiegler zeigen klinisch relevante Verfärbungen nach Anwendung der Plaquefärbelösung unter In-vitro-Bedingungen. Solche Verfärbungen konnten durch eine professionelle Zahnreinigung nicht entfernt werden. In der klinischen Praxis können Plaquefärbelösungen daher zu ästhetischen Defiziten bei mittels Glattflächenversieglern behandelten Zähnen führen. Die Auswirkungen von Plaquefärbelösungen unter klinischen Bedingungen (z. B. in Anwesenheit von Speichel, unterschiedliche Aspekte von jeweils aufgenommenen Nahrungsmitteln) sollten in klinischen Studien untersucht werden.

## Introduction

Enamel surface sealants are widely used in orthodontic practice to avoid enamel decalcifications in patients treated with fixed orthodontic appliances [6, 16, 21]. These sealants are indicated especially for patients with insufficient oral hygiene who are more likely to present with plaque and tooth stains. Dental plaque and stains on teeth with brackets require periodical removal by professional tooth cleaning (PTC). As a prophylactic measure during orthodontic treatment with fixed appliance, PTC should be performed every 3–6 months depending on the individual oral hygiene status [13].

Plaque disclosing solutions [3, 15, 18] are often used to support monitoring of the personal oral hygiene by the dentist, for instance, before performing PTC, or by patients to improve oral hygiene by self-checking the efficiency of daily tooth brushing [14].

A commonly used disclosing agent is the two-tone erythrosine-free disclosing dye solution Mira-2-Ton® (Hager & Werken, Duisburg, Germany), which can help to distinguish blue-dyed (brilliant blue FCF, E133, color index 42090) older plaque from pink-dyed (phloxine B, color index 45410) newer plaque [17, 18]. A possible staining effect of such disclosing solutions on orthodontic surface sealant cannot be excluded. However, so far only one study evaluated a possible staining effect of dyes used in disclosing solutions on dental materials. The authors found a very slight blue discoloration of resin composites after the use of a Colgate mouthwash, containing low concentrations of brilliant blue (E133) [7, 12]. Presently there are no studies on color changes of dental materials after the use of plaque disclosing solutions.

Orthodontic surface sealants, as we already recently showed in an in vitro study on four orthodontic surface sealants of different chemical compositions, are prone to discoloration by certain foods and beverages. Polishing with brush and prophy paste for 5 s reduced color changes; the original tooth color, however, could not be restored even after long polishing times (15 s) [5].

In particular in patients requiring frequent PTC involving the use of plaque disclosing solutions, the susceptibility of orthodontic surface sealants to discoloration could impair esthetics.

To our knowledge, there are currently no data available on the discoloration of orthodontic surface sealants after exposure of plaque disclosing solutions. Therefore, the aim of this study was to evaluate two different surface sealants for their discoloration by a plaque disclosing solution. Thus, the null hypothesis was that plaque disclosing solutions do not lead to a significant discoloration of orthodontic surface sealants or can be sufficiently removed by professional tooth cleaning.

## Methods

### Orthodontic surface sealants

Two frequently used surface sealants based on different chemical compositions were evaluated in this study: (1) the composite-based 18% filled sealant Pro Seal® (Opal Orthodontics, South Jordan, UT, USA, Lot No. 152740) and (2) the glass ionomer-based nanoparticle 38% filled sealant Opal® Seal™ (Reliance Orthodontic Products, Itasca, IL, USA, Lot No. BDBRJ).

### Sample preparation and group allocation

**Fig. 1:**
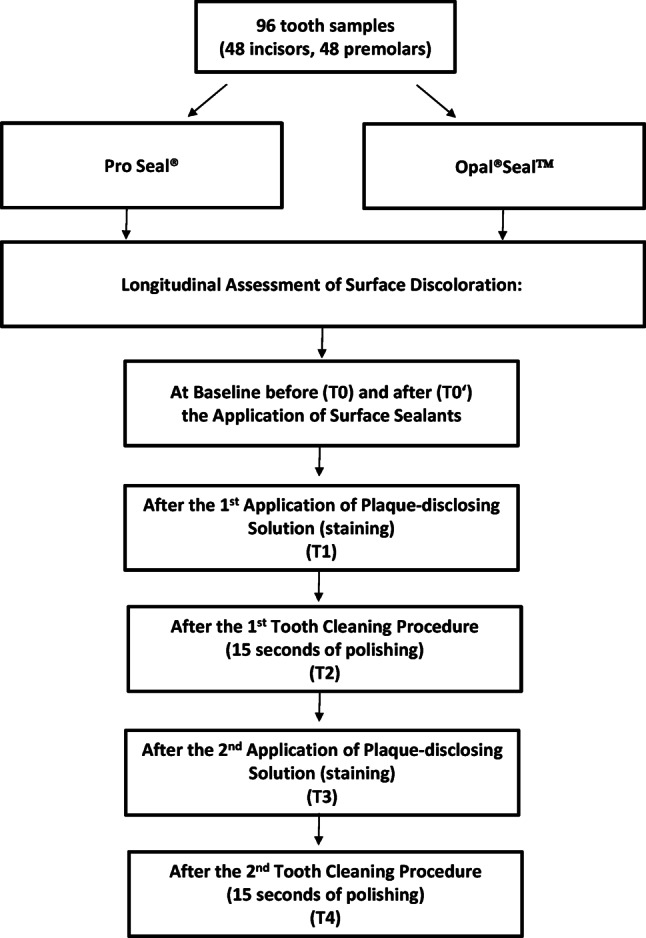
Workflow of the study

The workflow of the study is depicted in Fig. 1. In this in vitro study, 96 extracted lesion-free human teeth (48 incisors and 48 premolars) were randomly assigned to treatment with either Pro Seal® or Opal®Seal^TM^ (48 per group). Randomization was done manually by drawing teeth from an opaque container by a person without dental knowledge. Teeth crowns were separated using a diamond cutting disc (946.104.180 Komet Medical, Gebr. Brasseler GmbH & Co KG, Lemgo, Germany) and then embedded in silicone impression material (Silikon Knetmasse, Omnident Dental-Handelsgesellschaft GmbH, Germany).

All sealants were applied according to the manufacturers’ instructions. Light curing was performed using a bluephase® G2 polymerization lamp (Ivoclar Vivadent AG, Schaan, Liechtenstein); luminosity was routinely tested and found to be above 1200 mW/cm^2^.

### Application of disclosing solution

**Fig. 2:**
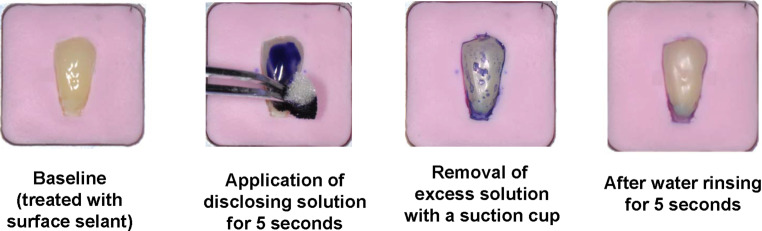
Application of plaque disclosing solution

Two-tone erythrosine-free plaque disclosing dye solution Mira-2-Ton ® (Hager & Werken, Duisburg, Germany) was used according to manufacturer’s instructions at room temperature (mean temperature 20 °C). Five seconds after application, excess plaque disclosure solution was removed with a suction cup and the tooth surface was rinsed with water for 5 s (Fig. 2). Hereafter the application of plaque disclosing solution will be referred to as “staining”.

### Color measurement

Color measurements were performed by use of a clinical spectrophotometer (Easyshade V, Vita Zahnfabrik, Bad Säckingen, Germany) which has been proven to be reliably suitable for laboratory and clinical use. To obtain high quality measurement, the device was centered on the geometric mid zone of the labial tooth surface and measurements were performed as triplicates on each tooth at each time point. Color data were collected and converted to L*, a*, and b* values according to the Commission Internationale de l’E’ clairage (CIE) by the results of intraoral spectrometer. Color differences (∆E) were calculated from L*, a*, and b* values using the following equation [2]:

$$\Updelta E_{ab}=\sqrt{\left(L_{1}-L_{2}\right)^{2}+\left(a_{1}-a_{2}\right)^{2}+\left(b_{1}-b_{2}\right)^{2}}$$

In the literature, different threshold values were discussed to describe clinically relevant changes, e.g., ∆E = 2 or ∆E = 2.72. Based on the study of Johnston et al. [8] and as in our previous work we used threshold value of ∆E = 3.7 [5].

### Polishing with brush and prophy paste

After each staining, polishing was performed at 2600 rotations/min with vertical loads of 1.5 N monitored with a precision balance according to Zimmer et al. [20]. Each polishing was done with a brush (Hawe Miniature Cleaning & Polishing Brushes, KerrHawe, Bioggio, Switzerland) and ready-made, fluoridated prophy paste (Cleanic®, KerrHawe, Bioggio, Switzerland, Lot No. 135145-09-’15) with low abrasiveness (RDA = 27) for 15 s using a low-speed handpiece (Sirona Dental System, Bensheim, Germany).

### Longitudinal assessment of surface color

Each sample was evaluated after the application of surface sealant (T0’, baseline), after the first staining (T1), after the first polishing for 15 s (T2), after the second staining (T3) and after the second polishing for 15 s (T4). We chose 15 s of polishing time because we showed in our previous work that color changes obtained after 15 s of polishing were more distinct than that after 5 s, albeit not significant [5].

### Statistics and methods

The sample size calculation was based on expected ∆E changes. As in our previous work we used a threshold value of ∆E = 3.77. Within this previous study, a standard deviation of 5.9 for ∆E was measured [5]. A ∆E threshold of 3.77, assuming a common standard deviation of 5.9 using a two-group test with a 0.05 two-sided significance level and a power of 0.80 yielded a sample size of 40 per group, we added 20% for potential dropouts. Thus, 48 teeth per group were evaluated.

Data from all investigations were collected and descriptive statistics was performed (mean, standard deviation). Changes in color after the above mentioned conditions were analyzed with the Mann–Whitney U test due to their skewed distribution. Two-sided *p* values <0.05 were considered statistically significant. No multiplicity adjustment was applied, and all *p* values should be interpreted descriptively. The data were processed using SigmaPlot 12.0 software (Systat Software, Inc., San Jose, CA, USA).

## Results

The main objective of this study was to measure the staining effect of the two-tone erythrosine-free plaque disclosing dye solution Mira-2-Ton ® on two orthodontic surface sealants longitudinally on extracted teeth.

### Clinically relevant (visible) color changes after application of plaque disclosing solution

**Fig. 3:**
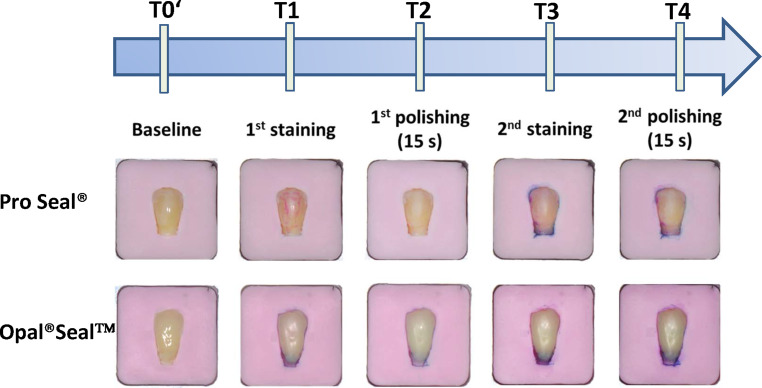
Exemplary sample photographs. Each sample was evaluated after the application of surface sealant (T0’, baseline), the first staining (T1), the first polishing for 15 s (T2), the second staining (T3) and second polishing for 15 s (T4). We found visible (clinically relevant) discoloration of sealant-treated tooth surfaces especially after the first staining of Pro Seal®-treated teeth (T1), and the second staining (T3) and second polishing (T4) of both sealant materials

Longitudinal color assessment revealed visible color changes, especially after the first staining of Pro Seal®-treated teeth (T1), and the second staining (T3) and second polishing (T4) of both surface sealants. Exemplary sample photographs are depicted in Fig. 3.

### Color changes of the individual surface sealants within the sealant group

**Fig. 4:**
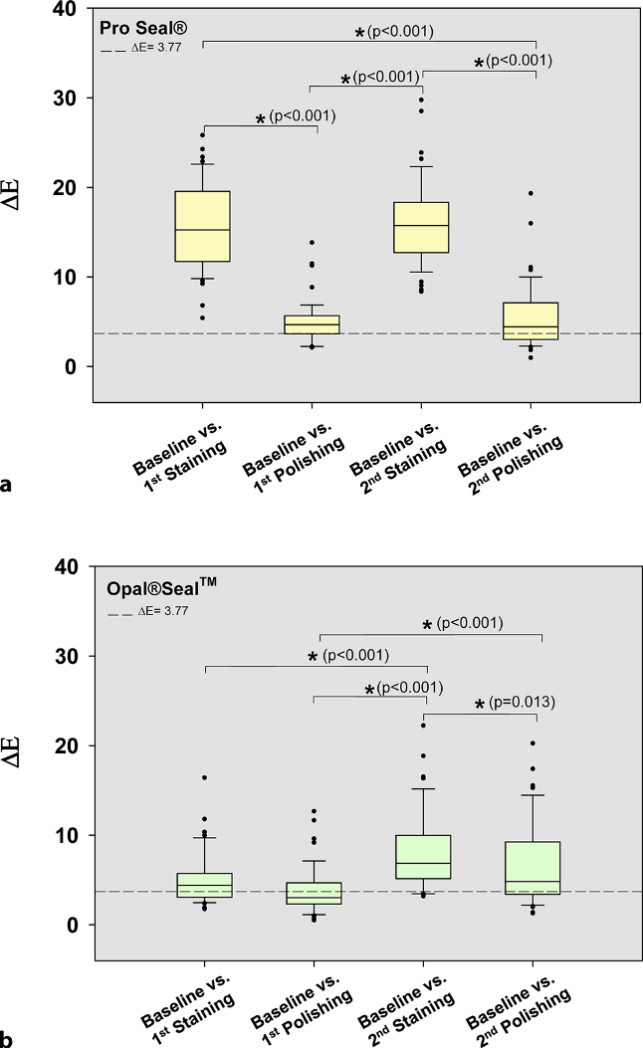
Color changes within the sealant group. **a** In Pro Seal®-treated teeth, ∆E values for T2 vs. T1, T3 vs. T2, T4 vs. T3 and T4 vs. T1 were statistically significant (*p < 0.05; Mann–Whitney U test). At all staining and polishing time points (T1–T4) color changes compared to baseline (T0’) were above the clinically relevant threshold ∆E = 3.77. **b** In Opal®Seal^TM^-treated teeth, ∆E values for T3 vs. T1, T3 vs. T2, and T4 vs. T2 were statistically significant (*p < 0.05; Mann–Whitney U test). Except for T2, the values were above the clinically relevant threshold value

The means of L*, a*, b* and ∆E values (±standard deviation) for baseline after the application of surface sealant and staining and polishing conditions on sealed tooth surfaces are given in the Table 1. Additionally, the mean color changes (∆E values) for each sealant group are depicted as box plots in Fig. 4.

**Table 1:** The means of L*, a*, b* and ∆E values (±standard deviation) for baseline after the application of surface sealant and staining and polishing conditions on sealed tooth surfaces

Material	Condition	L*	a*	b*	∆E (vs. baseline)
*Pro Seal® (n* *=48)*	Baseline	86.19 ± 4.15	1.62 ± 0.82	29.35 ± 3.2	0
	1^st^ staining	78.89 ± 4.04	10.38 ± 4.28	20.11 ± 4.03	15.57 ± 4.87
	1^st^ polishing	84.97 ± 3.99	2.31 ± 1.09	25.18 ± 3.10	4.96 ± 2.35
	2^nd^ staining	77.78 ± 3.69	6.67 ± 2.98	17.06 ± 3.56	16.17 ± 4.73
	2^nd^ polishing	84.23 ± 3.60	1.35 ± 1.40	25.43 ± 3.56	5.56 ± 3.63
*Opal®Seal*^*TM*^* (n* *=48)*	Baseline	84.76 ± 4.54	1.19 ± 0.74	29.51 ± 2.98	0
	1^st^ staining	81.58 ± 4.03	2.05 ± 1.27	26.24 ± 2.73	5.17 ± 2.92
	1^st^ polishing	83.21 ± 4.13	1.31 ± 0.79	26.95 ± 3.1	3.73 ± 2.59
	2^nd^ staining	79.66 ± 3.81	−0.08 ± 2.4	24.64 ± 2.66	8.11 ± 4.33
	2^nd^ polishing	82.12 ± 4.45	−0.85 ± 1.77	26.63 ± 2.78	6.61 ± 4.62

Clinically relevant threshold ∆E: 3.77

Staining of Pro Seal®-treated teeth caused clinically relevant changes of ∆E values. These were significantly reduced by polishing for 15 s after the first staining (T1 vs. T2) but remained above the clinically relevant threshold level. The second staining also caused significant increases of ∆E values which could be significantly reduced by polishing (T3 vs. T4); again ∆E values remained above the clinically relevant threshold level. Compared to baseline (T0’), color changes at all staining and polishing time points (T1–T4) were above the clinically relevant threshold level (∆E = 3.77) and caused visible esthetic deficits (Fig. 4a).

Overall, Opal®Seal^TM^-treated teeth exhibited a lesser degree of discoloration after both stainings than Pro Seal®-treated teeth. Polishing was not able to reduce color changes significantly at both time points (T1 vs. Ts and T3 vs. T4). However, due to the lesser extent of discoloration after the first staining, the first polishing was able to reduce ∆E values below the clinically relevant threshold (∆E = 3.77). Importantly, the degree of discoloration appeared to increase from the first to the second staining, and ∆E values were significantly higher after the second polishing compared to the first polishing. Taken together, as with Pro Seal®-treated teeth, compared to baseline (T0’) color changes at most staining and polishing time points (T1, T3 and T4) were above the clinically relevant threshold level. Opal®Seal^TM^-treated teeth revealed a lesser degree of discoloration compared with Pro Seal®-treated teeth but the significant increase of ∆E values from T2 (after first polishing) to T4 (after second polishing) suggests that there might be a progressive increase of discoloration for Opal®Seal^TM^-treated teeth which can no longer be reduced by polishing to reach levels below the clinically relevant threshold (Fig. 4b).

### Comparison of color changes between the two surface sealants

**Fig. 5:**
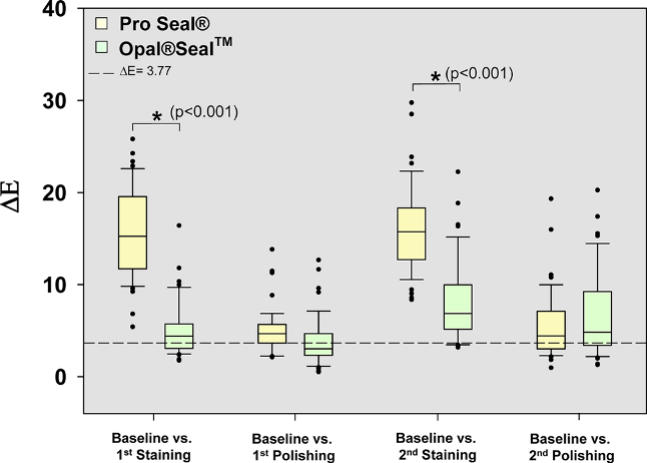
Comparison of color changes between the sealant groups. ∆E values of Pro Seal®- or Opal®Seal^TM^-treated teeth were significantly different after each staining vs. baseline. After the first polishing ∆E values were reduced below the clinically relevant threshold (∆E = 3.77) in Opal®Seal^TM^-treated teeth, but not in Pro Seal®-treated teeth. Interestingly, after second polishing ∆E values of Opal®Seal^TM^-treated teeth remained higher than those of Pro Seal®-treated teeth (*p < 0.05; Mann–Whitney U test)

For comparison the mean color changes (∆E values) for both surface sealants are depicted as box plots in Fig. 5.

Significant differences of mean ∆E values were detected between both sealants at both staining time points. Compared to Pro Seal®-treated teeth, Opal®Seal^TM^-treated teeth showed less discoloration after the application of disclosing solution. Unlike Pro Seal®-treated teeth discoloration of Opal®Seal^TM^-treated teeth could be reduced below clinically relevant levels by polishing. The second staining caused a discoloration on Pro Seal®-treated teeth that was comparable to the one after the first staining. In contrast, the second staining on Opal®Seal^TM^-treated teeth caused significantly more discoloration than the first staining. Moreover, after the second polishing, ∆E values of Opal®Seal^TM^-treated teeth were slightly higher than those of Pro Seal®-treated teeth, suggesting a progressive increase of discoloration of Opal®Seal^TM^-treated teeth which cannot be reduced by polishing.

## Discussion

This in vitro study aimed at mirroring the clinical setting as closely as possible. The study was performed using the experience of research group with the standardized preparation of tooth samples as well as with the application and durability of orthodontic surface sealants and the use of the plaque disclosing solution as a staining agent from our previous studies [4, 5, 16]. A clinically approved intraoral spectrophotometer of high reliability was used for the in vitro and in vivo measurements of color changes [9, 10, 19].

Orthodontic surface sealants are prone to discoloration, for instance, by foods and beverages [5]. In high-risk orthodontic patients wearing fixed appliances with low compliance, such surface sealants are frequently used as a preventive measure to avoid tooth demineralization and white spot lesions. Due to their low compliance, these patients require regular professional tooth cleaning, which is often preceded by staining with a plaque disclosing solution. Such solutions, however, contain relatively high concentrations of various plaque staining dyes which might lead to discoloration of the orthodontic surface sealants causing esthetic impairments. We have therefore evaluated the discoloration of the popular orthodontic surface sealants Pro Seal® and Opal®Seal^TM^ by the commonly used two-tone erythrosine-free disclosing dye solution Mira-2-Ton® in vitro on extracted teeth. In addition, we investigated whether Mira-2-Ton®-dependent discoloration of Pro Seal® and Opal®Seal^TM^ can be removed by polishing with brush and prophy paste during professional tooth cleaning. During treatment with fixed appliances, professional tooth cleaning is usually performed several times. Thus, in order to detect possible changes in the staining behavior of the sealers over time, we performed staining and polishing twice.

Both surface sealants, after the first staining, showed clinically relevant, visible discoloration. The extent of discoloration, however, was significantly higher in Pro Seal®-treated teeth. Polishing reduced the color changes of Pro Seal®-treated teeth significantly but discoloration remained clinically relevant. Discoloration of Opal®Seal^TM^-treated teeth was only slightly reduced by polishing; however, due to the lower initial discoloration, color changes were lowered below the clinically relevant threshold. The second staining and polishing of Pro Seal®-treated teeth was comparable with the results of the first staining. In contrast, Opal®Seal^TM^-treated teeth, after the second staining and polishing, showed significantly higher discoloration than after the first staining and polishing so that, as with Pro Seal®-treated teeth, clinically relevant discolorations remained. Our observations therefore showed that the extent of discoloration increased progressively on Opal® Seal^TM^-treated teeth.

In vitro, plaque disclosing solution caused discoloration of surface sealant-treated teeth which could not be sufficiently removed by professional tooth cleaning; thus, the null hypothesis was rejected.

A possible reason for the observed increase of discoloration of Opal® Seal^TM^ could be due to the different composition of the surface sealants. While Pro Seal® has a filler content of 18%, Opal®Seal^TM^ is a 38% filled sealant. Premaraj et al. compared the mechanical properties of Pro Seal® and Opal®Seal^TM^ by performing scanning electron microscope analyses and optical profilometer measurements. Opal®Seal^TM^ was found to contain filler particles (>250 nm) more than 2.5-fold the size of the particles in Pro Seal® (<100 nm). Polishing with brush and prophy paste of Opal®Seal^TM^ also caused high wear. This was explained by the authors through the abrasive contribution of the larger particles released by polishing of Opal®Seal^TM^. This high abrasion leads to an increased surface roughness due to the loss of relatively large filler particles, which might have contributed to the increased discoloration by the plaque disclosing solution observed after polishing of Opal®Seal^TM^ surfaces.

In addition to surface roughness, penetration depths of the plaque disclosing dyes could have also contributed to changes in the discoloration properties observed for Opal®Seal^TM^. We previously showed that abrasion from polishing of Pro Seal® and Opal®Seal^TM^ with brush and prophy paste were comparable (about 2–3 µm/s at vertical loads of 250 N) [16]. Abrasion was also comparable at vertical loads of 150 N used in this study, albeit smaller (0.5–0.8 µm/s, data not shown).

Since the abrasion of both surface sealants was comparable, it is likely that after the first polishing the penetration depth of the plaque disclosing dye is higher in Opal®Seal^TM^ than in Pro Seal®, so that abrasion during polishing for 15 s was too small to completely remove the stained layers of Opal®Seal^TM^ to significantly reduce color changes. The penetration depth of plaque disclosing dyes, thus, appears to be larger than that of coffee and red wine which occurs in the superficial layer (depth <20 μm) and can easily be removed by polishing [1].

Despite the observed reduction of color changes after the polishing, staining of both surface sealants was still visible. This should be considered when plaque staining is to be performed on teeth treated with these widely used orthodontic surface sealants. At the same time, it should be taken into account that the increased polishing required by the staining from plaque disclosing solutions can lead to further wear and thus to a loss of function of the surface sealants.

However, it must also be clearly stated here that the in vitro studies carried out here have numerous limitations with regard to the natural situation of the oral cavity and the transferability to the situation in the patient can therefore not be completely given. For instance, we cannot make any statement about the extent to which saliva and the proteins [11] it contains could affect the staining by plaque disclosing solutions. This also holds true for the potential impacts of nutrition, e.g., by cold or hot drinks, fruit juices or lemonades with acidic pH or abrasive foods. Furthermore, we have also not tested the influence of daily dental hygiene on discoloration of surface sealant-treated teeth by plaque disclosing solutions.

Thus, clearly further in vivo studies are necessary to fully investigate the extent of discoloration by plaque disclosing solutions and polishing dependent wear of orthodontic surface sealants.

## Conclusions

Surface sealants show clinically relevant discoloration after exposure to plaque disclosing solution under in vitro conditions. Such discolorations could not be removed by professional tooth cleaning. Thus, in clinical practice plaque disclosing solutions might cause esthetic deficits in surface sealant-treated teeth, the impact of plaque disclosing solutions under clinical conditions (e.g., in the presence of saliva and by various aspects of a person’s nutrition) should be investigated in clinical studies.

In the present in vitro investigation the following conclusions can be drawn with respect to discoloration of surface sealants.

- Both Opal®Seal^TM^ and Pro Seal® showed significant and clinically relevant discoloration after staining with a plaque disclosing solution.
- Polishing was not sufficient to reduce discoloration to clinically irrelevant values.
- In vitro, plaque disclosing solutions caused discoloration of surface sealant treated teeth; we suggest that this might be taken into account when using them in vivo.
